# Synthetic mRNA is a more reliable tool for the delivery of DNA-targeting proteins into the cell nucleus than fusion with a protein transduction domain

**DOI:** 10.1371/journal.pone.0182497

**Published:** 2017-08-14

**Authors:** Ivan Leontovyc, David Habart, Sarka Loukotova, Lucie Kosinova, Jan Kriz, Frantisek Saudek, Tomas Koblas

**Affiliations:** 1 Department of Experimental Medicine, Institute for Clinical and Experimental Medicine, Prague, Czech Republic; 2 Department of Diabetes, Institute for Clinical and Experimental Medicine, Prague, Czech Republic; Louisiana State University Health Sciences Center, UNITED STATES

## Abstract

Cell reprogramming requires efficient delivery of reprogramming transcription factors into the cell nucleus. Here, we compared the robustness and workload of two protein delivery methods that avoid the risk of genomic integration. The first method is based on fusion of the protein of interest to a protein transduction domain (PTD) for delivery across the membranes of target cells. The second method relies on de novo synthesis of the protein of interest inside the target cells utilizing synthetic mRNA (syn-mRNA) as a template. We established a Cre/lox reporter system in three different cell types derived from human (PANC-1, HEK293) and rat (BRIN-BD11) tissues and used Cre recombinase to model a protein of interest. The system allowed constitutive expression of red fluorescence protein (RFP), while green fluorescence protein (GFP) was expressed only after the genomic action of Cre recombinase. The efficiency of protein delivery into cell nuclei was quantified as the frequency of GFP^+^ cells in the total cell number. The PTD method showed good efficiency only in BRIN-BD11 cells (68%), whereas it failed in PANC-1 and HEK293 cells. By contrast, the syn-mRNA method was highly effective in all three cell types (29–71%). We conclude that using synthetic mRNA is a more robust and less labor-intensive approach than using the PTD-fusion alternative.

## Introduction

Cell reprogramming is an emerging approach for treating an increasing number of human diseases [1]. Reprogramming factors, such as transcription factors, need to be delivered effectively into target cell nuclei. Delivery methods based on viral vectors or transposon systems are highly effective [2,3]. However, they carry inherent risks of unpredictable modifications of the target cell genome by random and irreversible integrations of exogenous DNA, which can cause insertional mutagenesis and carcinogenesis. Therefore, such approaches are not suitable for eventual clinical applications [4,5].

To avoid this limitation, alternative integration-free strategies have been developed. Direct application of recombinant proteins to cells is generally not feasible because most proteins do not cross cellular membranes. However, specialized protein domains that naturally facilitate transmembrane transport of polypeptides have been discovered [6] and harnessed as a novel protein delivery tool [7]. Dohoon et al. [8] successfully used a protein-based protocol to generate induced pluripotent stem cells (iPS), albeit with a lower efficiency in comparison to virus-based protocols [9]. Another promising strategy relies on the *de novo* synthesis of cargo proteins inside the target cell, where the structural information is provided by synthetic mRNA [10,11]. Warren et al. used this approach to successfully reprogram somatic cells into iPS, and subsequently to terminally differentiated myogenic cells [12]. To the best of our knowledge, although a number of delivery methods have been compared [13], a direct comparison between the two integration-free methods utilizing either the protein transduction domain (PTD) or synthetic mRNA has not been performed.

The aim of the present study was to provide such a comparison using diverse cell lines. The focus of our laboratory is the reprogramming of cells of pancreatic origin [14]. We selected the human pancreatic cancer cell line PANC-1 [15], which was previously used for cell fate manipulation and reprogramming using other methods [16], and the rat insulinoma cell line BRIN-BD11 [17], which represents terminally differentiated cells with regulated secretory pathways. Additionally, we chose the human embryonic kidney cell line HEK293 [18], which is of neuronal origin [19] and has been used extensively for producing exogenous proteins in research and industry [20]. Cre recombinase is an enzyme not normally present in mammalian cells. It has the capacity to specifically rearrange nuclear DNA in conjunction with the targeting sequence loxP [21]. The delivery of Cre recombinase to cell nuclei can be unequivocally detected by monitoring phenotypic effects of the irreversible, site-specific recombination of genomic DNA, such as small deletions [22, 23]. Using Cre recombinase as a model of the cargo protein, we designed and prepared PTD- and mRNA-based Cre recombinase constructs. We engineered three Cre-sensitive cell lines utilizing green fluorescent (GFP) and red fluorescent proteins (RFP) as the reporter system. Using this model, we compared the efficiency, reliability, and the workload of the two respective methods.

## Materials and methods

### Experimental design

**Fig 1:**
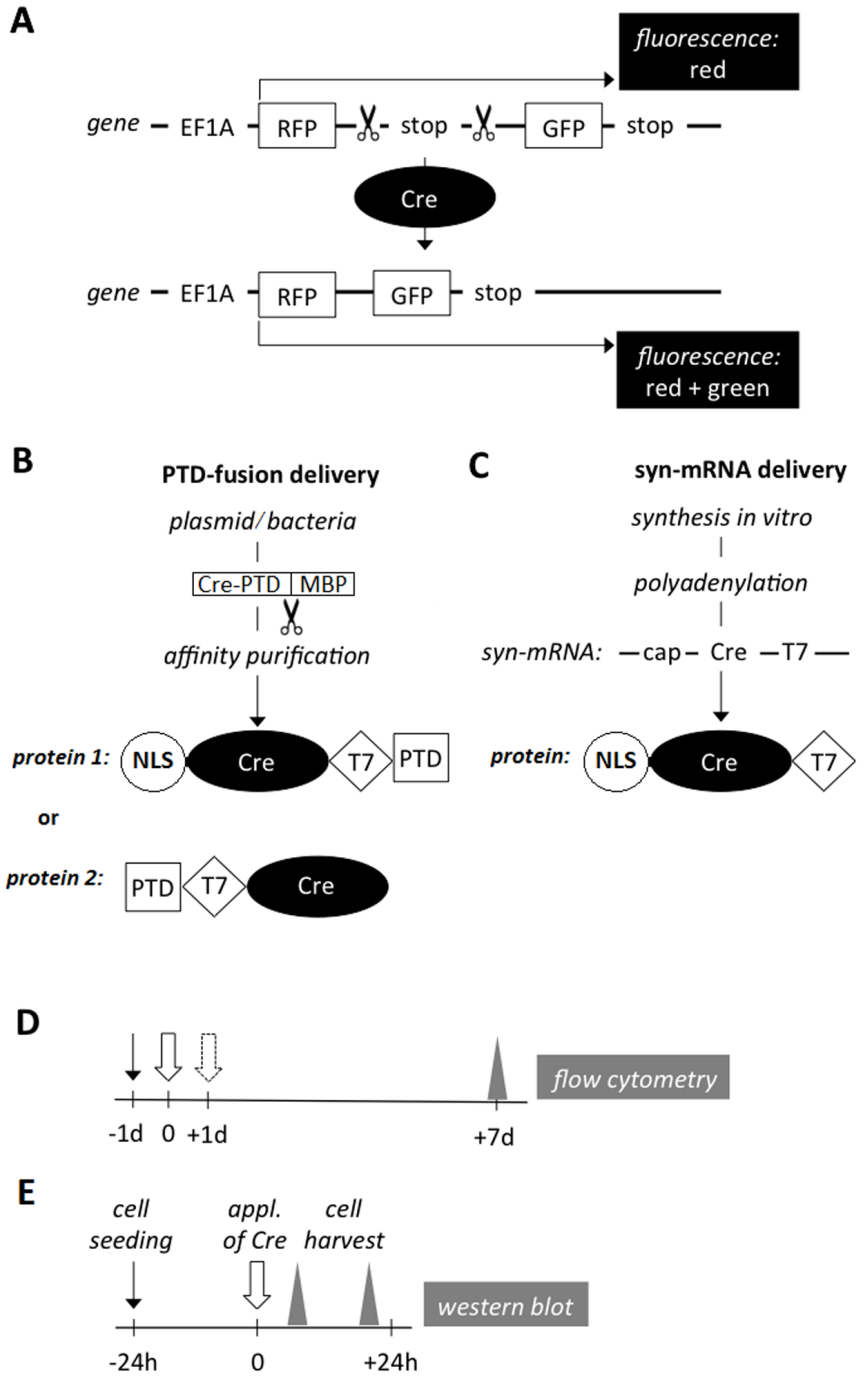
Experimental design. (A) RFP/GFP expression cassette inserted randomly into the genomes of the target cells. In these Cre-sensitive cells, the GFP expression was dependent on the delivery of functional Cre protein into cell nuclei. Scissors, parallel loxP sequences; Cre, Cre recombinase; stop, two stop codons. (B) The PTD-fusion proteins were produced in bacteria and purified in three steps. (C) The syn-mRNA (cap-NLS-T7-Cre) was synthesized *in vitro*. aa, number of amino acids; NLS, Nuclear localization signal (9 aa); Cre, Cre recombinase (343 aa); PTD, Protein transduction domain of HIV TAT (11 aa); T7, T7-tag (11 aa). Time frames of the flow cytometry (D) and western blot (E) analyses. Black arrows, cell seeding; white arrows, administration of Cre recombinase; dashed white arrow, second administration of Cre recombinase; grey triangles, harvesting of cells for analysis.

Three cell lines were genetically modified using a DNA expression cassette that encoded red and green fluorescent proteins placed downstream of a strong constitutive promoter (Fig 1A). Coding sequences of RFP and GFP were separated by two stop codons flanked by two parallel recognition sites for Cre recombinase (Fig 1A, S1 Fig). Constitutively expressed RFP was used to prepare Cre-responsive cell clones. Cre recombinase-sensitive expression of GFP was used to detect the activity of the recombinase delivered into the cell nuclei. 2A self-cleaving peptide was employed to separate RFP and GFP from a bicistronic product (Supplementary information 1) [24]. Two delivery methods were tested: the purified recombinant fusion protein (PTD-Cre, Fig 1B) and the synthetic mRNA construct (syn-mRNA-Cre, Fig 1C). The efficiency of the Cre protein delivery was quantified using flow cytometry (Fig 1D). The amount of intracellular Cre protein was compared by western blot (Fig 1E).

### Generation and culture of Cre-responsive cell lines

An expression cassette was designed (Fig 1A, S1 Fig) consisting of an RFP-loxP-stop-loxP-GFP sequence under the EF1a promoter and the hygromycin resistance selection marker. This was cloned into the piggyBac vector pD557-RA (DNA2.0, Menlo Park, CA) between the restriction sites *Bmt*I *and Bam*HI. A total of 3 μg of the piggyBac construct was combined with TransIT-X2 (Mirus, Madison, WI) at a 1:2 ratio and added to the respective cell lines PANC-1, BRIN-BD11 and HEK293 (all from Sigma-Aldrich, St. Louis, MO). After 14 days, cells were detached with 0.63% trypsin (Sigma-Aldrich). Clonal populations of the genetically modified cells were obtained by the sorting of single-cell suspensions to one cell per well, based on the RFP fluorescence signal using a BD Influx Cell sorter (Becton Dickinson, Franklin Lakes, NJ). The Cre-responsive cells were given the names fl-PANC, fl-BRIN and fl-HEK.

Cells were cultured in ventilated flasks (Corning, Corning, NY) at 37°C, atmospheric O_2_ and 5% CO_2_. The PANC-1 cells were cultured in Dulbecco’s modified Eagle’s medium (Sigma-Aldrich) supplemented with 10% fetal bovine serum (FBS), 25 U/ml penicillin, 25 μg/ml streptomycin, 1 mM L-glutamine, and 1% Glutamax. The BRIN-BD11 cells were cultured in RPMI-1640 medium (Sigma-Aldrich) supplemented with 10% FBS, 25 U/ml penicillin, 25 μg/ml streptomycin, 1 mM L-glutamine, and 1% Glutamax. HEK293 cells were cultured in MEM medium supplemented with 10% FBS (both from Sigma-Aldrich), 25 U/ml penicillin, 25 μg/ml streptomycin, 1 mM L-glutamine, 1% Glutamax, and 1% non-essential amino acids solution (all from Thermo Fisher Scientific, Waltham, MA).

### Production of PTD-Cre recombinase protein

Two variants of PTD-Cre protein were prepared in a native state (Fig 1B). Correct folding of Cre recombinase was ensured by its fusion to the maltose binding protein (MBP), which functioned as a molecular chaperone [25]. MBP was subsequently cleaved off by TEV protease and removed by purification. The design of PTD-Cre1 (S2 Fig) was based on AAV-pgk-Cre, a kind gift from Patrick Aebischer (Addgene plasmid # 24593). The design of PTD-Cre2 (S3 Fig) was based on pTAT-Cre [26] (Addgene plasmid # 35619). The respective DNA constructs were cloned into the pMALc5x plasmid (New England Biolabs, Ipswitch, MA) between the restriction sites *Sac*I and *Bam*HI using the In-fusion HD Cloning Kit (Clontech, Mountain View, CA). NEB express competent *E*. *coli* (New England Biolabs) were transformed with the plasmids and cultured in LB medium (Carl Roth, Karlsruhe, DE) with 2% glucose (Sigma-Aldrich) and 50 μg/ml ampicillin (Serva, Heidelberg, DE) at 37°C, and agitated at 260 rpm. Protein expression was induced by 0.17 mM IPTG (Sigma-Aldrich) for 4 hours at 30°C, after which the bacterial cells were sonicated in a MBP-binding buffer containing 50 μg/ml DNase (Roche, Rotkreuz, CH) and 1 mg/ml lysozyme (Serva). The supernatant was placed on a MBPTrapHP column (GE Healthcare Life Science, Little Chalfont, UK) with MBP-specific affinity, and the purified MBP-PTD-Cre-T7 protein was eluted. Next, the MBP was cleaved off by the addition of TEV protease and separated from PTD-Cre-T7 by a second round of the MBPTrapHP column purification. Finally, PTD-Cre-T7 protein was quantified using the BCA protein assay (Thermo Fisher Scientific) and transferred to the fresh culture media using a 10 kDa Amicon filter (Merck Millipore, Darmstadt, DE). The PTD-Cre protein aliquots (53 μM) were stored at -20°C for one month.

### Production of syn-mRNA-Cre

The syn-mRNA-Cre construct (Fig 1C) was synthesized *in vitro* using the T7 mScript Standard mRNA Production System (CELLSCRIPT, Madison, WI) and 2 μg of purified DNA template. The template DNA was designed (S4 Fig) and synthesized using AAV-pgk-Cre, a kind gift from Patrick Aebischer (Addgene plasmid # 24593). A custom ribonucleotide blend comprised of 3′-0-Me-m7G(5′)ppp(5′)G ARCA cap analog, pseudouridine triphosphate, 5-methylcytidine triphosphate (TriLink Biotechnologies, San Diego, CA), ATP, and GTP (New England Biolabs) was prepared. The final reaction mixture (20 μL), containing 6 mM ARCA cap analog, 3.0 mM ATP, and 1.5 mM of each of the other nucleotides, was incubated for 1 hour at 37°C. The DNA template was then degraded by Turbo DNase (Life Technologies, Grand Island, NY), which was removed by ammonium acetate precipitation. The residual 5′-triphosphates were degraded by 2 hour incubation at 37°C with Antarctic phosphatase (New England Biolabs), which was removed by ammonium acetate precipitation. After a 2 hour treatment at 37°C with yeast Poly(A) Polymerase (Affymetrix, Santa Clara, CA), the polyadenylated synthetic mRNA was finally repurified with a MEGAclear Transcription Clean-Up Kit, diluted with RNAsecure Resuspension Solution and quantified with a Qubit fluorometer (all from Thermo Fisher Scientific).

### Administration of PTD-Cre and syn-mRNA-Cre

The Cre-responsive cell lines were grown for several days in their respective culture media, which were changed at various degrees of confluence (to account for subsequent growth) prior to the addition of PTD-Cre or syn-mRNA-Cre. The purified PTD-Cre protein, originally dissolved in the respective culture media, was added directly to the cells at three final serial dilutions (15, 7.5 and 3.75 μM). The syn-mRNA-Cre was added at three final decimal dilutions (2.1, 0.21, and 0.021 nM) in Lipofectamine/Opti-MEM transfection reagent. Lipofectamine messenger MAX transfection reagent was first diluted with Opti-MEM medium at a 1:33 volume ratio. Then, syn-mRNA-Cre diluted in Opti-MEM (all from Thermo Fisher Scientific) was added at a 1:1 volume ratio. In several experiments the administration of the protein or the ribonucleic acid was repeated after 24 hours (Fig 1D). A full description of these protocols can be found here: dx.doi.org/10.17504/protocols.io.h7jb9kn

### Western blot

Western blot analysis was performed on fully-confluent Cre-responsive cell lines harvested from 24-well plates 6 or 22 hours after the single administration of PTD-Cre (15 nM) or syn-mRNA-Cre (2.1 nM). The cells were lysed using RIPA buffer composed of 150 mM NaCl, 1% IGEPAL CA-630, 0.5% sodium deoxycholate, 0.1% SDS, and 50 mM Tris, pH 8.0 [27]. Cell lysates (14 μg total protein per well) were mixed with 4x Laemmli loading buffer containing 8% SDS, 40% glycerol, 0.02% bromophenol blue, 250 mM Tris, and 20% 2-mercaptoethanol (all from Sigma-Aldrich), pH 6.8, heated at 95°C for 3 min, and run on a 15% polyacrylamide gel and transferred to PVDF membranes (Merck Millipore) using a Pierce G2 electroblotter (Thermo Fisher Scientific). The membranes were blocked with 3% BSA (Sigma-Aldrich). Primary antibodies included a rabbit anti-T7 antibody (Abcam, Cambridge, UK) for detecting Cre recombinase (1:2000 dilution) and a mouse anti-beta-actin antibody (Sigma-Aldrich) as a loading control (1:7500 dilution). The secondary antibodies included goat anti-rabbit IgG-HRP (Merck Millipore) and rabbit anti-Mouse IgG-HRP (Thermo Fisher Scientific), each diluted 1:50000. Chemiluminescent SuperSignal West Dura Extended Duration Substrate (Thermo Fisher Scientific) was used for detection. The signals were acquired using a G:BOX Chemi XR5 (Syngene, Cambridge, UK).

### Fluorescence microscopy

Fluorescence microscopy was performed on Cre-responsive cell lines six days after a single administration of PTD-Cre (15 nM) or syn-mRNA-Cre (2.1 nM) to 10% confluent culture. The cells were cultured on untreated glass coverslips in 48-well culture plates (Sigma-Aldrich) and then fixed with 4% formaldehyde (Polysciences, Warrington, PA). The cell nuclei were counterstained with 4’,6-diamidine-2’-phenylindole dihydrochloride (DAPI) (Thermo Fisher Scientific). Stained cover slips were mounted on slides with Mowiol mounting medium. Cell samples were imaged using an EVOS FL Auto Cell Imaging System (Thermo Fisher Scientific).

### Flow cytometry

Cre-responsive cell lines at 10% confluence were treated with a single and double administration of PTD-Cre or syn-mRNA-Cre using the three above-mentioned concentrations. Six days later, the cells were detached from the flat bottoms of 48-well plates (area 0.95 cm^2^) using 0.63% trypsin (both from Sigma-Aldrich). Single-cell suspensions were washed and stored at 4°C in PBS buffer for up to 2 hours and analyzed using a BD LSRII analyzer (Becton Dickinson). A total of 5000–10000 events were counted for each sample. The respective untreated cells were used as negative controls for gating.

### Data evaluation and statistics

All experiments were carried out independently in triplicate, and the results are expressed as the mean ± standard deviation (SD). GraphPad Prism 5 was used to construct asymmetrical (five-parameter) dose-response curves and to calculate two-tailed unpaired Student’s *t*-tests. P-values <0.05 were considered statistically significant.

## Results and discussion

### Cre-responsive cell lines

**Fig 2:**
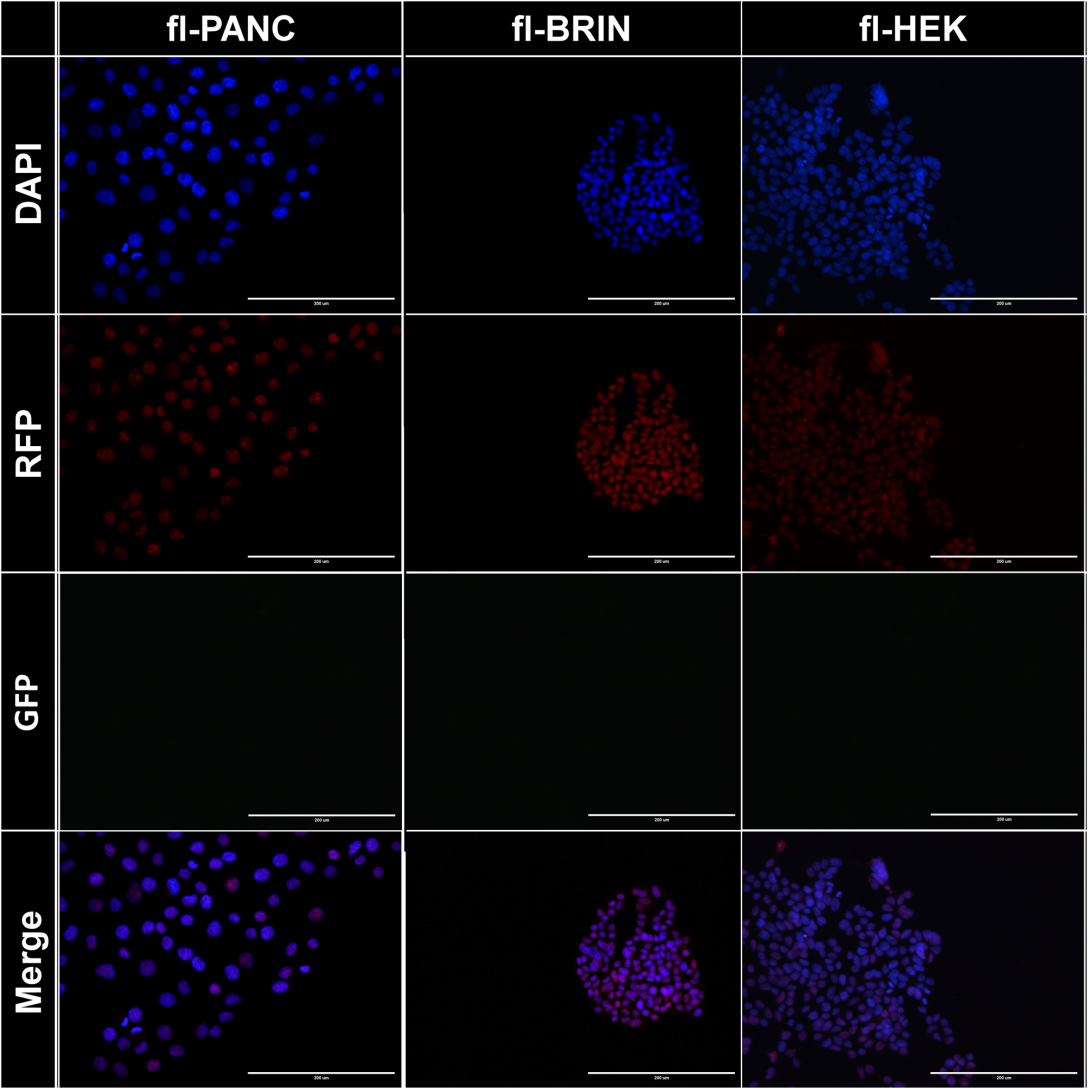
Verification of engineered clones after their expansion. No leaky GFP expression was observed.

Cre-responsive cell lines were created from the original cell lines PANC-1, BRIN-BD11 and HEK293 by the genomic insertion of the expression cassette shown in Fig 1A and specified in S1 Fig, followed by single-cell sorting to produce clonal populations. After the sorting, approximately 20–30 of the 96 wells contained RFP-positive cells, depending on the original cell lines. For each cell line, three clones were expanded and preserved. A single clone of each line was then used throughout the study. After expansion, the presence of the construct was verified using fluorescence microscopy (RFP positivity). Theoretically possible inadvertent GFP expression in the absence of Cre recombinase (leakage) was excluded by the absence of a green fluorescence signal in any of the clones (Fig 2).

### Detection of active Cre recombinase delivery into cell nuclei

**Fig 3:**
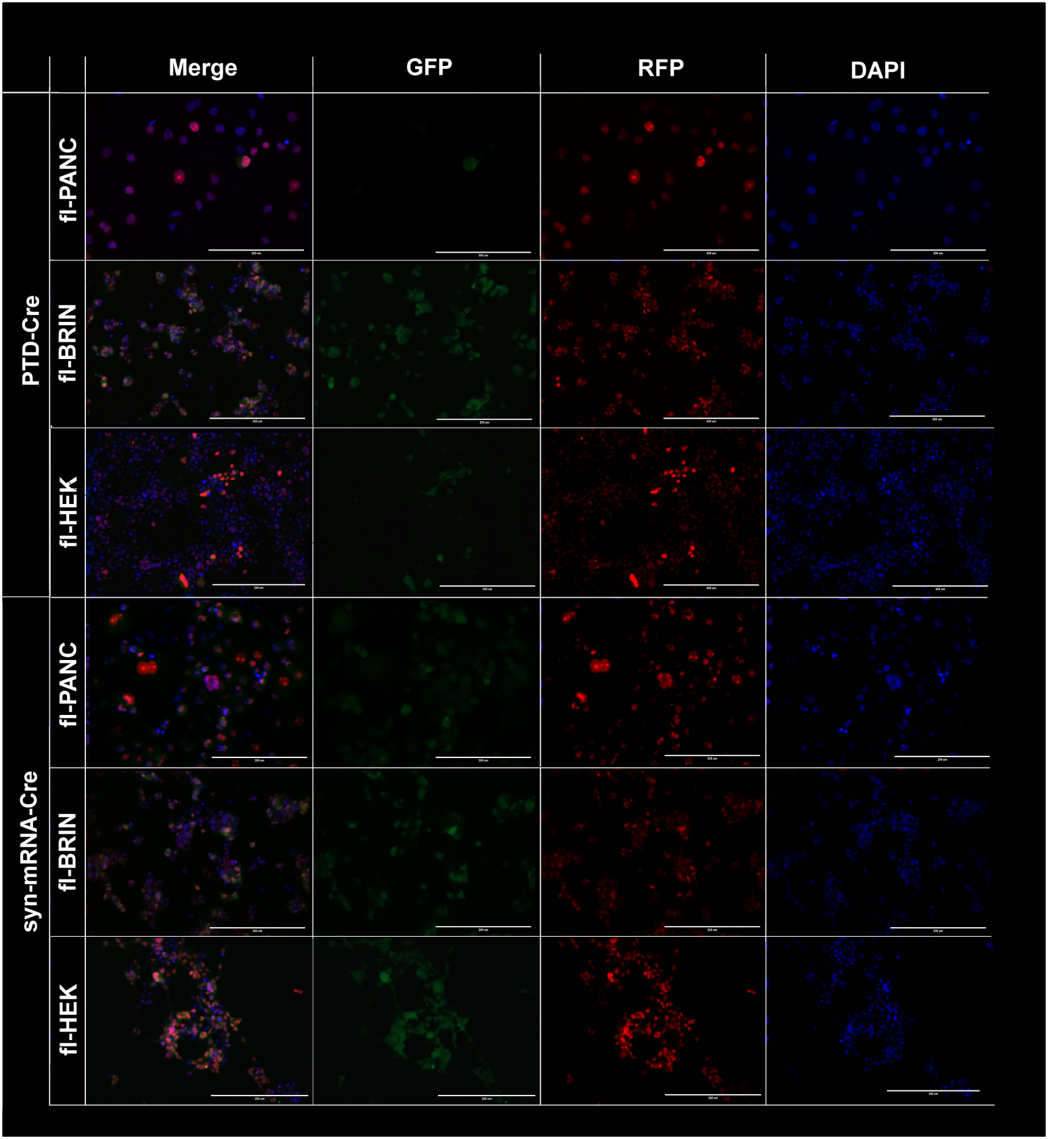
Detection of Cre recombinase activity in cell nuclei of the target cells after administration of PTD-Cre (15 μM) or syn-mRNA-Cre (2.1 nM). Bar 200 μm.

Correct folding of the PTD-Cre fusion protein was assured by the chaperone action of the Maltose-Binding protein [28], which was encoded by the expression vector. There are a variety of PTD sequences [29], but predicting the best one for a particular cell type is not possible. Our preliminary experiments using TAT3-GFP and TAT8-GFP constructs suggested that the latter penetrated into PANC-1 cells better, and it was therefore used throughout the study. Cre-responsive cells grown on glass coverslips were treated with a single administration of PTD-Cre1 (15 μM) or syn-mRNA-Cre (2.1 nM) for 24 hours. Successful delivery of a functional Cre protein into the cell nuclei was verified microscopically by detecting the Cre-mediated synthesis of GFP in the cytoplasm of individual cells. The GFP signal became visible three days after treatment, irrespective of the cell type (fl-PANC, fl-BRIN, fl-HEK) or the delivery method. The signal reached a maximum around day 6 and maintained an apparently unchanged level for another 7 days (data not shown). The number of positive cells clearly differed between the two delivery strategies and among the cell types. While approximately half of the syn-mRNA-treated cells from each cell line produced GFP, only a small fraction of cells was GFP-positive after treatment with PTD-Cre1. The best result quantified by flow cytometry was 0.123±0.067% (n = 3) of GFP^+^ cells in fl-BRIN cells (data not shown). This failure occurred despite the presence of the nuclear localization sequence (NLS) in the PTD-Cre1 protein (S2 Fig). Spontaneous entry of Cre recombinase into the cell nucleus has been previously reported [30]. We modified our original construct accordingly by changing the domain order and removing the NLS (Fig 1B, S3 Fig). Using this PTD-Cre2 construct at the highest concentration (15 μM), the number of GFP-positive fl-BRIN cells increased, but there was no substantial change among the fl-PANC and fl-HEK cells (Fig 3). PTD-Cre2 was used in subsequent experiments.

### Efficiency of Cre protein delivery into the cell nuclei

**Fig 4:**
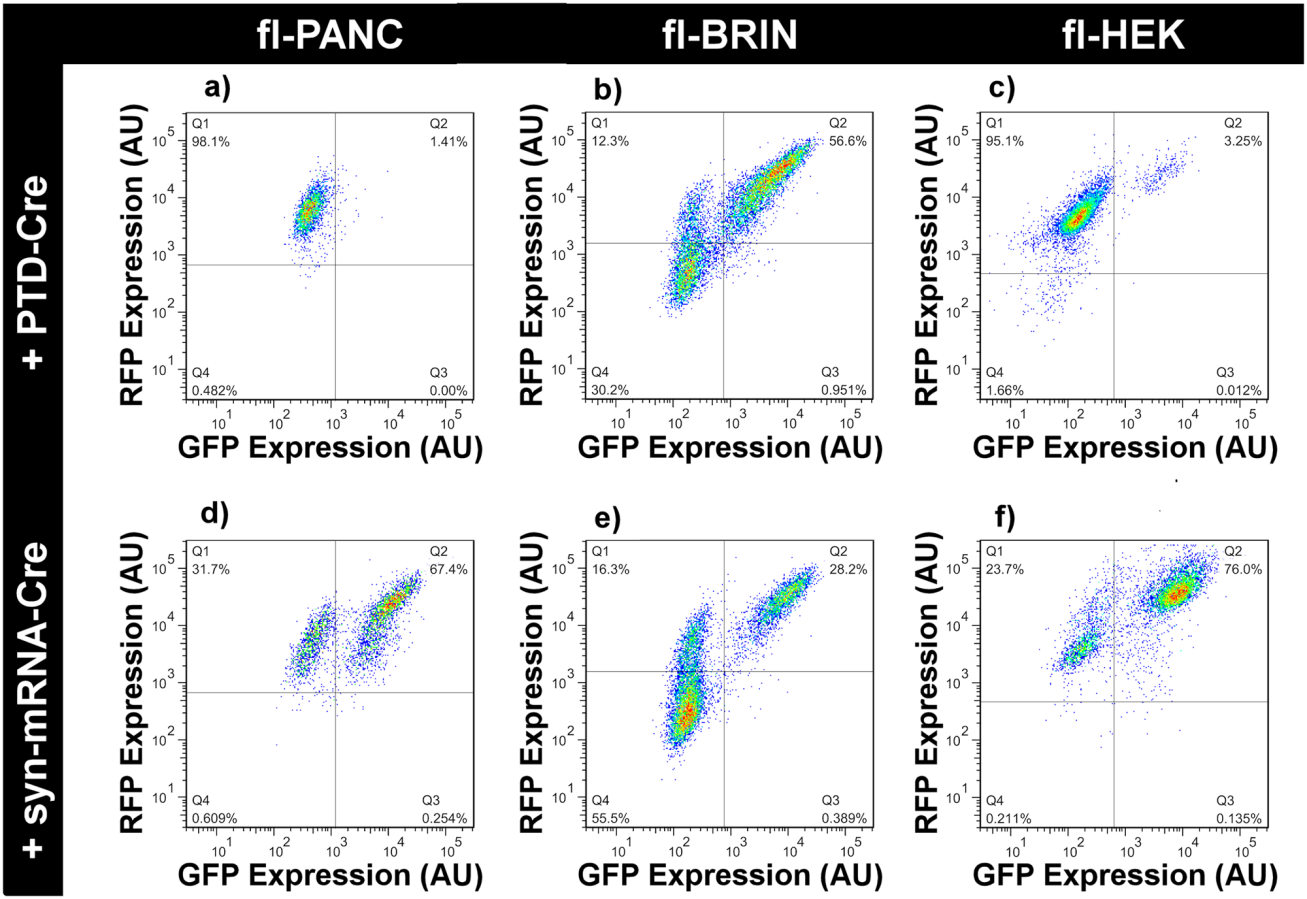
Flow cytometry scatter plots obtained on day 7 after single administration of 15 μM PTD-Cre (a-c) or 2.1 nM syn-mRNA-Cre (d-f) in fl-PANC (a,d), fl-BRIN (b,e), and fl-HEK (c,f) cells.

The Cre-responsive cells were grown on plastic dishes (area 0.95 cm^2^) and treated with either PTD-Cre2 or syn-mRNA-Cre at low (10%) confluence to account for their expansion over a period of one week. Six days after the first administration (Fig 1D), the cells were harvested in a single-cell suspension and the GFP^+^ cells were quantified using flow cytometry. Fig 4 depicts representative scatter plots obtained from each cell type after administration of either PTD-Cre2 (15 μM) or syn-mRNA-Cre (2.1 nM).

Direct comparison of the methods employing substances of different classes (protein vs. mRNA) was realized by calculating the individual doses relative to the maximum dose achievable for each substance. Protein precipitation limited the maximum protein concentration of PTD-Cre2 to 15 μM. It was practical to further increase the dose by repeated administration of this concentration (no toxicity was observed). The dose of syn-mRNA-Cre turned out to be limited by cell toxicity. After the double administration of 2.1 nM of syn-mRNA-Cre, dead cells appeared in the medium and the growth of the remaining attached cells was reduced. Double administration of lower concentrations had no visible toxic effects (data not shown). To prevent this innate cell toxicity, we used modified nucleotides [31, 32]. However, our syn-mRNA was not HPLC-purified. Such purification would reduce cytotoxic byproducts of the *in vitro* mRNA synthesis and might potentially allow for the use of even higher concentrations of syn mRNA-Cre [33].

**Fig 5:**
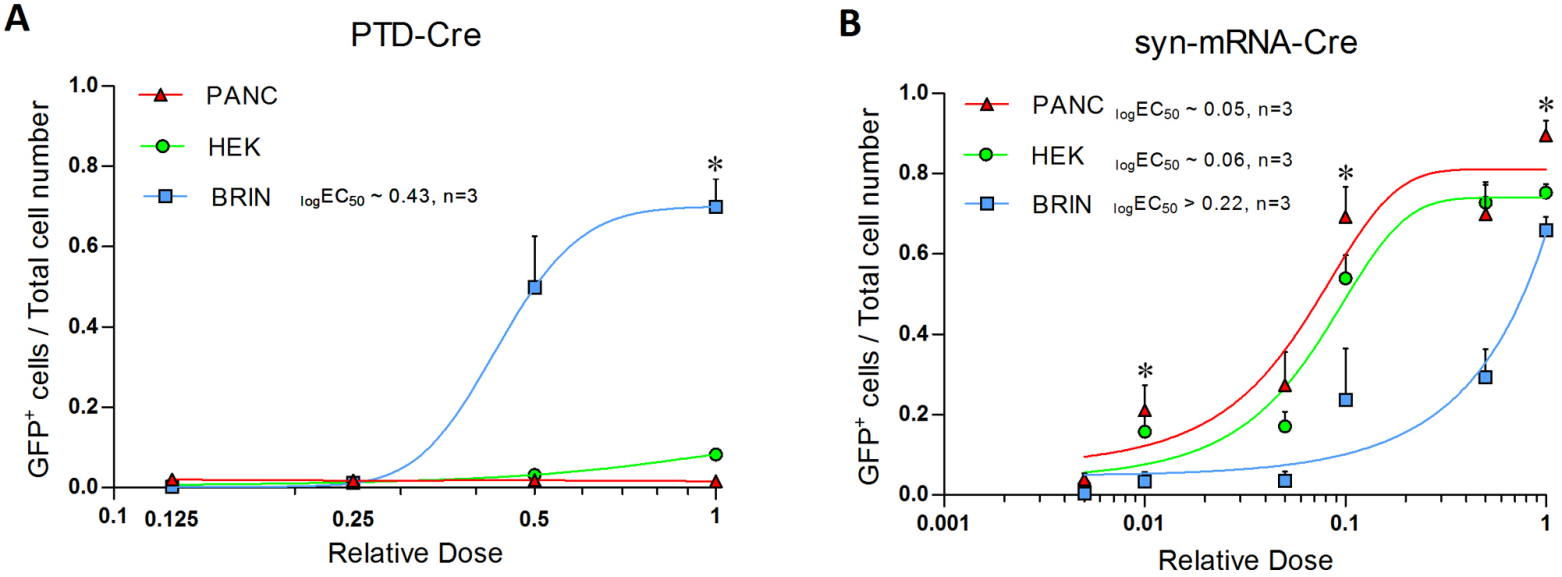
Frequency of GFP^+^ cells after the treatment of Cre-responsive cells with either PTD-Cre2 (A) or syn-mRNA-Cre (B). Analysis of flow cytometry data from three independent experiments. X-axis: relative dose on the logarithmic scale, (1 is the maximum dose); Asterisk, double administration; Y-axis: efficiency of the delivery of Cre recombinase into nuclei of fl-PANC (red triangles), fl-BRIN (blue squares), fl-HEK (green circles) cells; Error bars: mean±SD, n = 3; GraphPad was used to construct asymmetrical (five-parameter) dose-response curves and to calculate the approximate logEC50.

The frequencies of successful delivery of Cre recombinase protein into cell nuclei by day seven after treatment with either PTD-Cre2 protein or syn-mRNA are summarized in Fig 5 for three independent experiments in the Cre-responsive cell lines. PTD-Cre2 mediated an appreciable delivery of an active Cre recombinase protein only in the nuclei of fl-BRIN cells (Fig 5A). Syn-mRNA-Cre was successful in all three cell types (Fig 5B). In the fl-PANC and fl-HEK cells (but not in fl-BRIN cells), the highest (toxic) dose was observed at the plateau of the dose-response curve (Fig 5B). Half maximum effective doses (EC50) were calculated as non-toxic doses at which the efficiencies of both delivery methods could be directly compared across the cell types. Table 1 demonstrates that PTD-Cre2 failed to deliver functional Cre recombinase into the nuclei of two out of three cell types, while the robustness of syn-mRNA-Cre was demonstrated by its substantial efficacy irrespective of cell type.

**Table 1:** Comparison of PTD-fusion and syn-mRNA. Half maximum effective doses (EC50) and half maximum efficiency in three cell types.

	Half Effective dose (EC50)		Half maximum efficiency	
	PTD-Cre (nmol/cm^2^)	syn-mRNA-Cre (pmol/cm^2^)	PTD-Cre (%)	syn-mRNA-Cre (%)
PANC	failed	0.047	failed	0.39
BRIN	2.71	0.205	0.38	0.24
HEK	failed	0.058	failed	0.37

**Fig 6:**
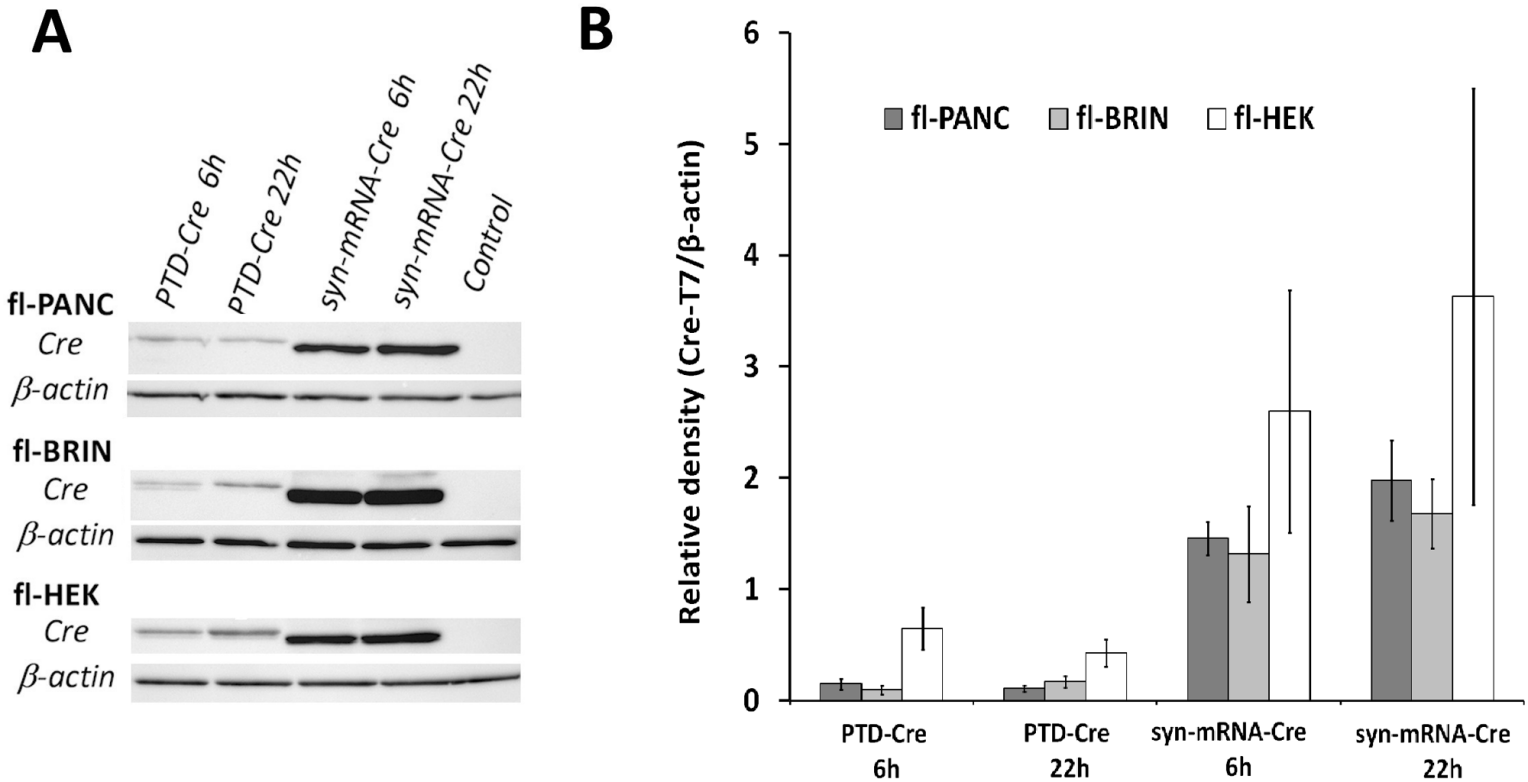
Quantification of intracellular Cre recombinase protein in three cell types at two time points after single administration of either 15 μM PTD-Cre or 2.1 nM syn-mRNA-Cre. (A) Western blot images are representative of three independent experiments. (B) Relative quantification of Cre recombinase protein, mean±SD, *n=3*.

The two tested methods deliver the cargo protein inside the cells by different means. Therefore, differential robustness of the two methods could potentially be explained by different amounts of the Cre protein entering the treated cells. To determine the amounts of Cre protein that entered the cells, the Cre-responsive cells were treated with either PTD-Cre2 or syn-mRNA-Cre (single administration of the maximum concentrations) and harvested 6 or 22 hours later for western blot analysis. Fig 6 shows at each time point that the relative amount of Cre protein in cell homogenates was 4–19-fold higher in the syn-mRNA-treated cells than in the PTD-Cre2-treated cells. It is noteworthy, however, that the Cre protein in the PTD-treated fl-BRIN cells was 3–7 times lower than in the fl-HEK cells (p-values were 0.016 and 0.049 after 6 and 22 hr incubations, respectively), although the Cre-mediated recombination was successful in the former and failed in the latter. Using the PTD-Cre in fl-HEK cells, we obtained results similar to those previously published [27]. Further clarification of the observation that the intracellular level of the cargo protein did not correspond with its nuclear effect is beyond the scope of this manuscript.

### Workload

Starting from the transformed bacteria, the preparation of the PTD-Cre protein was labor-intensive (Materials and methods) and took two full working days, yielding a total of 2–3 mg of protein from 1 L of culture. The treatment of cells in one well (0.95 cm^2^) with the highest concentration required 0.1 mg of protein. Starting with the ready-made DNA template, the preparation of syn-mRNA-Cre took up to 8 h, yielding approximately 60 μg of the syn-mRNA-Cre. The treatment of cells in one well (area 0.95 cm^2^) with the highest concentration required 0.2 μg of synthetic syn-mRNA-Cre. The *in vitro* synthesis of a specific synthetic mRNA required less time and effort than the multistep preparation of a purified recombinant protein.

## Conclusion

We conclude that in comparison to the PTD fusion-based protocol, the synthetic mRNA-based method is less cell type-dependent, less work-intensive, and more efficacious for protein delivery into cell nuclei. We recommend synthetic mRNA as a first-line approach, particularly when the cell type of interest has not been previously tested.

## Supporting information

S1 Fig Expression cassette, nucleotide sequence. (DOCX)

S2 Fig PTD-Cre1 nucleotide sequence. (DOCX)

S3 Fig PTD-Cre2 nucleotide sequence. (DOCX)

S4 Fig Template DNA sequence for syn-mRNA-Cre. (DOCX)
