# Supplementary material for: Synthetic mRNA is a more reliable tool for the delivery of DNA-targeting proteins into the cell nucleus than fusion with a protein transduction domain
Source: PLoS One. 2017 Aug 14;12(8):e0182497. doi: 10.1371/journal.pone.0182497 (PMC5555570; doi:10.1371/journal.pone.0182497)
Supplement: S1 Fig — (DOCX) [file pone.0182497.s001.docx]

**Supporting information 1**

Expression cassette –diagram and nucleotide sequence:


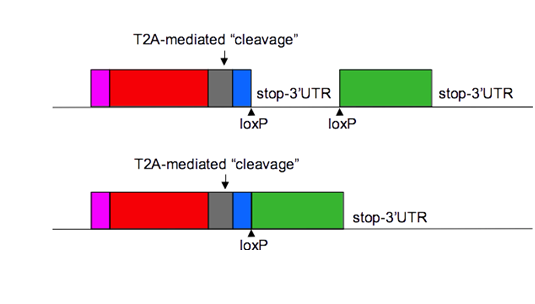


**Figure 1.**The expression cassette prior and after the Cre-mediated recombination. T2A-mediated separation of RFP and GFP polypetides.

EF1a promoter, NLS, RFP protein,T2A peptide sequence, V5 tag, loxP, double stop codon,3´UTR and polyadenylation signal sequence,loxP, GFP protein, 3´UTR

cgtgaggctccggtgcccgtcagtgggcagagcgcacatcgcccacagtccccgagaagttggggggaggggtcggcaattgaaccggtgcctagagaaggtggcgcggggtaaactgggaaagtgatgtcgtgtactggctccgcctttttcccgagggtgggggagaaccgtatataagtgcagtagtcgccgtgaacgttctttttcgcaacgggtttgccgccagaacacaggtaagtgccgtgtgtggttcccgcgggcctggcctctttacgggttatggcccttgcgtgccttgaattacttccacgcccctggctgcagtacgtgattcttgatcccgagcttcgggttggaagtgggtgggagagttcgaggccttgcgcttaaggagccccttcgcctcgtgcttgagttgaggcctggcctgggcgctggggccgccgcgtgcgaatctggtggcaccttcgcgcctgtctcgctgctttcgataagtctctagccatttaaaatttttgatgacctgctgcgacgctttttttctggcaagatagtcttgtaaatgcgggccaagatctgcacactggtatttcggtttttggggccgcgggcggcgacggggcccgtgcgtcccagcgcacatgttcggcgaggcggggcctgcgagcgcggccaccgagaatcggacgggggtagtctcaagctggccggcctgctctggtgcctggcctcgcgccgccgtgtatcgccccgccctgggcggcaaggctggcccggtcggcaccagttgcgtgagcggaaagatggccgcttcccggccctgctgcagggagctcaaaatggaggacgcggcgctcgggagagcgggcgggtgagtcacccacacaaaggaaaagggcctttccgtcctcagccgtcgcttcatgtgactccacggagtaccgggcgccgtccaggcacctcgattagttctcgagcttttggagtacgtcgtctttaggttggggggaggggttttatgcgatggagtttccccacactgagtgggtggagactgaagttaggccagcttggcacttgatgtaattctccttggaatttgccctttttgagtttggatcttggttcattctcaagcctcagacagtggttcaaagtttttttcttccatttcaggtgtcgtgaATGGCACCCAAAAAGAAACGTAAAGTGTCTCTATCCAAACAGGTCCTCCCACGGGACGTCAAAATGCGTTACCACATGGACGGCTGTGTCAATGGTCATCAGTTCATCATCGAAGGCGAAGGTACTGGCAAACCGTACGAGGGGAAGAAAATCTTGGAGCTAAGAGTGACGAAGGGCGGTCCTCTGCCCTTTGCATTCGATATCCTGTCCAGCGTGTTCACTTATGGAAACAGGTGCTTTTGTGAGTACCCCGAGGACATGCCGGATTACTTCAAACAGAGCCTGCCCGAGGGACATAGCTGGGAACGAACGCTCATGTTCGAGGATGGAGGCTGCGGCACCGCGTCTGCTCACATCTCACTGGACAAAAACTGCTTCGTGCACAAGAGCACTTTCCACGGTGTGAACTTCCCAGCAAACGGTCCGGTGATGCAGAAGAAAGCCATGAACTGGGAGCCCTCGTCCGAACTGATCACCGCATGCGATGGAATCCTTAAGGGGGATGTAACCATGTTCCTGCTGCTGGAGGGCGGTCACAGACTCAAGTGTCAGTTCACTACCTCATACAAAGCCCACAAAGCGGTGAAGATGCCACCGAATCACATCATCGAGCATGTACTCGTCAAAAAGGAGGTCGCGGACGGCTTCCAAATCCAAGAGCATGCAGTGGCAAAACATTTCACAGTGGACGTGAAAGAAACCGGCACATCTGAGGGCAGGGGAAGTCTGCTAACATGCGGGGACGTGGAGGAAAATCCCGGCCCCATGCTAGCGGGCAAACCGATCCCGAATCCACTGCTGGGTCTGGACTCTACCATAACTTCGTATAATGTATGCTATACGAAGTTATCGTGAGTAAGTAATTGAGATCGCTTTCCTGCTTCGTCCAATAATTATGGGGAGACACTTCTTTTTGTAACTTGAAAAATTTGTATTACAGAATAAAATGTATTTAGGAGACACTTTCAATAAAACATCTTTATTTTCATTACAACTGTGTGTTGGTTTTTTTGTCTGCCCCCCACCCTTGCCGTCTGCGCCGTTTAAAAATCAAAGGGGTTAGATAACTTCGTATAATGTATGCTATACGAAGTTATCGATGACTGCCCTGACCGAAGGTGCTAAGCTGTTTGAGAAGGAGATTCCGTACATCACCGAGCTGGAAGGGGACGTCGAAGGAATGAAGTTCATCATCAAGGGAGAAGGAACCGGGGACGCTACGACTGGAACCATTAAGGCCAAGTATATCTGTACCACTGGAGATCTGCCAGTGCCTTGGGCCACCCTTGTGTCAACCCTCTCGTATGGAGTGCAGTGTTTTGCTAAGTACCCTAGCCACATTAAGGACTTCTTCAAATCCGCCATGCCGGAAGGTTATACCCAAGAGCGCACCATTTCTTTTGAGGGAGATGGAGTGTACAAGACCCGCGCGATGGTCACCTATGAGAGGGGATCTATCTACAACCGGGTGACTCTGACTGGAGAAAACTTTAAGAAGGACGGGCATATTCTTCGGAAGAATGTCGCCTTCCAGTGCCCTCCCAGCATCCTTTACATTCTCCCCGACACTGTGAACAACGGAATCCGCGTGGAGTTCAATCAAGCCTACGACATCGAGGGGGTGACGGAGAAGCTGGTGACCAAGTGTAGCCAGATGAATCGGCCACTGGCCGGTTCAGCGGCTGTCCACATTCCGCGCTACCATCATATCACTTATCACACTAAGCTCTCCAAAGACCGCGATGAGAGGAGAGATCACATGTGCCTGGTGGAAGTGGTCAAGGCCGTCGATCTCGATACCTATCAGTAAGCTCGCTTTCTTGCTGTCCAATTTCTATTAAAGGTTCCTTTGTTCCCTAAGTCCAACTACTAAACTGGGGGATATTATGAAGGGCCTTGAGCATCTGGATTCTGCCTAATAAAAAACATTTATTTTCATTGC
