# Supplementary material for: Synthetic mRNA is a more reliable tool for the delivery of DNA-targeting proteins into the cell nucleus than fusion with a protein transduction domain
Source: PLoS One. 2017 Aug 14;12(8):e0182497. doi: 10.1371/journal.pone.0182497 (PMC5555570; doi:10.1371/journal.pone.0182497)
Supplement: S2 Fig — (DOCX) [file pone.0182497.s002.docx]

**Supporting information 2**

PTD-Cre1 nucleotide sequence:

TEV Protease cleaving site, NLS, Cre Recombinase, T7 tag, HIV-TAT PTD, His Tag

GAGAACCTGTACTTTCAGGGTATGGTGCCCAAGAAGAAGAGGAAAGTCTCCAACCTGCTGACTGTGCACCAAAACCTGCCTGCCCTCCCTGTGGATGCCACCTCTGATGAAGTCAGGAAGAACCTGATGGACATGTTCAGGGACAGGCAGGCCTTCTCTGAACACACCTGGAAGATGCTCCTGTCTGTGTGCAGATCCTGGGCTGCCTGGTGCAAGCTGAACAACAGGAAATGGTTCCCTGCTGAACCTGAGGATGTGAGGGACTACCTCCTGTACCTGCAAGCCAGAGGCCTGGCTGTGAAGACCATCCAACAGCACCTGGGCCAGCTCAACATGCTGCACAGGAGATCTGGCCTGCCTCGCCCTTCTGACTCCAATGCTGTGTCCCTGGTGATGAGGAGAATCAGAAAGGAGAATGTGGATGCTGGGGAGAGAGCCAAGCAGGCCCTGGCCTTTGAACGCACTGACTTTGACCAAGTCAGATCCCTGATGGAGAACTCTGACAGATGCCAGGACATCAGGAACCTGGCCTTCCTGGGCATTGCCTACAACACCCTGCTGCGCATTGCCGAAATTGCCAGAATCAGAGTGAAGGACATCTCCCGCACCGATGGTGGGAGAATGCTGATCCACATTGGCAGGACCAAGACCCTGGTGTCCACAGCTGGTGTGGAGAAGGCCCTGTCCCTGGGGGTTACCAAGCTGGTGGAGAGATGGATCTCTGTGTCTGGTGTGGCTGATGACCCCAACAACTACCTGTTCTGCCGGGTCAGAAAGAATGGTGTGGCTGCCCCTTCTGCCACCTCCCAACTGTCCACCCGGGCCCTGGAAGGGATCTTTGAGGCCACCCACCGCCTGATCTATGGTGCCAAGGATGACTCTGGGCAGAGATACCTGGCCTGGTCTGGCCACTCTGCCAGAGTGGGTGCTGCCAGGGACATGGCCAGGGCTGGTGTGTCCATCCCTGAAATCATGCAGGCTGGTGGCTGGACCAATGTGAACATTGTGATGAACTACATCAGAAACCTGGACTCTGAGACTGGGGCCATGGTGAGGCTGCTCGAGGATGGGGACGGTTCTGGCATGGCGTCTATGACCGGTGGCCAGCAAATGGGTTCTGGCAGGAAGAAGCGGAGACAGCGACGAAGAGGCCACCACCACCACCACCATCATTAA
