# Supplementary material for: Synthetic mRNA is a more reliable tool for the delivery of DNA-targeting proteins into the cell nucleus than fusion with a protein transduction domain
Source: PLoS One. 2017 Aug 14;12(8):e0182497. doi: 10.1371/journal.pone.0182497 (PMC5555570; doi:10.1371/journal.pone.0182497)
Supplement: S4 Fig — (DOCX) [file pone.0182497.s004.docx]

**Supporting information 4**

Template DNA sequence for syn-mRNA-Cre:

T7 promoter, Rat betaGlobin 5´UTR, NLS, Cre Recombinase, T7 tag, Human betaGlobin 3´UTR

TAATACGACTCACTATAGGGACATTTGCTTCTGACACAACTGTGTTCACTAGCAACCTCAAACAGCCACCATGGTGCCCAAGAAGAAGAGGAAAGTCTCCAACCTGCTGACTGTGCACCAAAACCTGCCTGCCCTCCCTGTGGATGCCACCTCTGATGAAGTCAGGAAGAACCTGATGGACATGTTCAGGGACAGGCAGGCCTTCTCTGAACACACCTGGAAGATGCTCCTGTCTGTGTGCAGATCCTGGGCTGCCTGGTGCAAGCTGAACAACAGGAAATGGTTCCCTGCTGAACCTGAGGATGTGAGGGACTACCTCCTGTACCTGCAAGCCAGAGGCCTGGCTGTGAAGACCATCCAACAGCACCTGGGCCAGCTCAACATGCTGCACAGGAGATCTGGCCTGCCTCGCCCTTCTGACTCCAATGCTGTGTCCCTGGTGATGAGGAGAATCAGAAAGGAGAATGTGGATGCTGGGGAGAGAGCCAAGCAGGCCCTGGCCTTTGAACGCACTGACTTTGACCAAGTCAGATCCCTGATGGAGAACTCTGACAGATGCCAGGACATCAGGAACCTGGCCTTCCTGGGCATTGCCTACAACACCCTGCTGCGCATTGCCGAAATTGCCAGAATCAGAGTGAAGGACATCTCCCGCACCGATGGTGGGAGAATGCTGATCCACATTGGCAGGACCAAGACCCTGGTGTCCACAGCTGGTGTGGAGAAGGCCCTGTCCCTGGGGGTTACCAAGCTGGTGGAGAGATGGATCTCTGTGTCTGGTGTGGCTGATGACCCCAACAACTACCTGTTCTGCCGGGTCAGAAAGAATGGTGTGGCTGCCCCTTCTGCCACCTCCCAACTGTCCACCCGGGCCCTGGAAGGGATCTTTGAGGCCACCCACCGCCTGATCTATGGTGCCAAGGATGACTCTGGGCAGAGATACCTGGCCTGGTCTGGCCACTCTGCCAGAGTGGGTGCTGCCAGGGACATGGCCAGGGCTGGTGTGTCCATCCCTGAAATCATGCAGGCTGGTGGCTGGACCAATGTGAACATTGTGATGAACTACATCAGAAACCTGGACTCTGAGACTGGGGCCATGGTGAGGCTGCTCGAGGATGGGGACGGTTCTGGCATGGCGTCTATGACCGGTGGCCAGCAAATGGGTTAGAGCGCTCGCTTTCTTGCTGTCCAATTTCTATTAAAGGTTCCTTTGTTCCCTAAGTCCAACTACTAAACTGGGGGATATTATGAAGGGCCTTGAGCATCTGGATTCTGCCTAATAAAAAACATTTATTTTCATTGCATGCTATACACGTATGCGAGAACAAAAAAAAAAA
